# Nationwide variation of snakebite incidence in Kenya: Community surveys as an integrated NTD approach

**DOI:** 10.1371/journal.pntd.0013732

**Published:** 2025-11-21

**Authors:** George O. Oluoch, Wyckliff Omondi, Cecilia Ngari, Nicholas R. Casewell, Steven A. Wasonga, Florence Wakesho, Titus Waititu, Dickson Kioko, Agnes Kithinji, Tonny O. Ngage, Loreta A. Asila, Stanley Parkurito, Geoffrey M. Kephah, Ezekiel Adino, Daniel Lang’at, Sarah P. Olalo, Irene Chami, Abdinasir Amin, Martin M. Josphat, Mary Amuyunzu-Nyamongo, Sultani H. Matendechero, Peter G. Mwethera, Frank-Leonel Tianyi, Robert A. Harrison, David G. Lalloo, Shelui Collinson, Ymkje Stienstra

**Affiliations:** 1 Kenya Snakebite Research and Intervention Centre, Kenya Institute of Primate Research, Ministry of Health, Nairobi, Kenya; 2 Centre for Snakebite Research and Interventions, Liverpool School of Tropical Medicine, Liverpool, United Kingdom; 3 Vector Borne and Neglected Tropical Diseases Unit, Ministry of Health, Nairobi, Kenya; 4 Fred Hollows foundation, Nairobi, Kenya; 5 END FUND, Nairobi, Kenya; 6 Amref Health Africa, Nairobi, Kenya; 7 African Institute for Health and Development, Nairobi, Kenya; 8 Ministry of Health, Nairobi, Kenya; 9 University of Groningen, Department of Internal Medicine/Infectious Diseases, University Medical Centre Groningen, Groningen, The Netherlands; Fundação de Medicina Tropical Doutor Heitor Vieira Dourado: Fundacao de Medicina Tropical Doutor Heitor Vieira Dourado, BRAZIL

## Abstract

**Introduction:**

Snakebite envenoming is a neglected tropical disease (NTD) of public health concern in Kenya. Its true burden remains elusive with an over-reliance on health facility-based data and geographically limited community surveys. This study aimed to generate data on snakebite burden in Kenyan communities and to capture the variation in incidence rate across the country by integrating snakebite incidence questions into nationwide Mass Drug Administration (MDA) campaigns for other NTDs.

**Methods:**

A cross-sectional community survey was conducted, nested within MDA campaigns targeting trachoma, schistosomiasis and soil transmitted helminths. Data collection spanned from July 2022 to August 2023. Incidence rates per 100,000 person-years were calculated, and correlation between snakebite incidence and population density was assessed. Community survey data were compared to the reported snakebite cases in health facilities by the Kenya Health Information System (KHIS).

**Results:**

A total of 13,117,754 individuals from 17 counties participated in the MDA surveys, representing 27.6% of Kenya’s total population. Across these counties, 4,667 snakebite cases were reported over the previous year, with a slightly higher incidence rate among males (39.3 cases per 100,000 inhabitants) compared to females (32.2 cases per 100,000 inhabitants). County-level incidence rates varied, with Turkana County reporting the highest incidence rate (412.9 cases per 100,000 inhabitants) and Vihiga County recording the lowest (3.7 cases per 100,000 inhabitants). Discrepancies existed between health facility attendance reported by community members and numbers reported by KHIS.

**Conclusion:**

Integration of snakebite data collection with MDA campaigns allowed rapid and highly cost-effective data capture from a quarter of Kenya’s population. The community data demonstrated considerable variation in incidence rates and discrepancies with hospital-based data. This informs resource allocation for treatment and prevention and emphasizes the need for robust integrated approaches to assess the burden of snakebite envenoming both in health facilities and communities.

## Author summary

Snakebite envenoming is a neglected tropical disease posing a significant health risk in Kenya, yet its true impact remains unclear due to limited data from community surveys and over-reliance on health facility-based data. To address this gap, snakebite-related questions were embedded into nationwide Mass Drug Administration (MDA) campaigns targeting other neglected tropical diseases such as trachoma, schistosomiasis, and soil-transmitted helminths. Between July 2022 and August 2023, over 13 million people across 17 counties—representing 27.6% of Kenya’s population—participated in the MDA. A total of 4,667 snakebite cases were reported, with slightly higher rates among males. Incidence varied widely between counties, from 412.9 cases per 100,000 in Turkana to just 3.7 in Vihiga. Community-reported health facility visits did not align with official records from the Kenya Health Information System. This approach demonstrated that integrating snakebite surveillance into existing public health campaigns allowed rapid and cost-effective data capture from a quarter of Kenya’s population. The findings support resource allocation for treatment and prevention.

## Introduction

Snakebite envenoming (SBE) is a neglected tropical disease (NTD) of major public health concern, particularly in tropical regions where environmental, geographical, and demographic factors intersect to elevate the risk of human-snake encounters [1]. Despite increasing global awareness, the true burden of SBE, especially in low- and middle-income countries (LMICs), remains elusive, hindering effective resource allocation and public health interventions [1,2].

In Africa, the estimated incidence of snakebite is alarmingly high, at one million bites per year [3], suggesting that the continent accounts for a substantial portion of global snakebite cases. Annual snakebite estimates vary from 100 to 650 bites per 100,000 inhabitants with mortality rates exceeding 10 deaths per 100,000 [4]. Factors such as rural living, agricultural activities, and limited access to healthcare exacerbate the risk of SBE in many African communities, however poor data availability complicates efforts to accurately assess the true extent of the problem.

Unravelling the true extent of SBE presents a complex challenge. Relying solely on health facility data offers only a partial view, that fails to capture the full spectrum of SBE’s impact on public health [5,6]. Given widespread underreporting, inadequate SBE surveillance systems and limited healthcare accessibility, particularly in rural areas, this narrow approach leads to a significant underestimation of the burden faced by affected communities, masking the broader socio-economic and health implications of SBE. This lack of comprehensive data impedes the development of effective intervention strategies, exacerbating the consequences of SBE in vulnerable populations. Addressing the challenge of quantifying SBE requires a comprehensive approach that overcomes the systemic barriers to accurate reporting and healthcare access in at-risk regions.

The challenges of poor data availability and limited resources are not unique to SBE but are common across the spectrum of NTDs. Given the impact this has for addressing NTD burden, the World Health Organization (WHO), in the strategy ‘*A Road Map for Neglected Tropical Diseases 2021-2030’* [7] has called for integration across programmes in order to “improve [their] cost-effectiveness, coverage and geographical reach”. By consolidating efforts and resources for programme management, intervention delivery and disease monitoring, there is the potential to efficiently tackle the burden of multiple diseases simultaneously, maximizing impact and cost-effectiveness. Moreover, by collaborating across diseases that share characteristics, a more holistic approach to public health interventions becomes possible, emphasizing common preventive measures, promoting and improving treatment accessibility, and fostering community engagement.

Within this context, Kenya, situated in eastern Africa, faces a high burden from SBE and reported incidence and mortality figures underscore the urgency of addressing this issue. Annual incidence rates as high as 150 cases per 100,000 inhabitants have been reported in a community-based survey conducted in Kilifi County [8], though the vast majority of available Kenyan data still come from health facility reporting. A facility-based study conducted in the same county showed rates of just 44 cases per 100,000 inhabitants [9]. Another study conducted in Samburu County found a one year mortality rate of 28 deaths per 100,000 inhabitants [10]. Several other NTDs are also endemic in Kenya, and over the years considerable progress has been made in controlling several of these conditions, including lymphatic filariasis, onchocerciasis, trachoma, schistosomiasis, and soil-transmitted helminths (STH) [11]. Mass drug administration (MDA) is a public health intervention whereby a preventive chemotherapy is dispensed to all individuals within a defined population, irrespective of their disease status. Preventive chemotherapy, distributed through MDA, is one of the main interventions used to control and eliminate these NTDs [12]. With extensive coverage across the country [13] such platforms facilitate the collection of comprehensive data on disease prevalence and distribution, providing an opportunity to streamline resource allocation and delivery.

In light of this, our research sought to integrate snakebite surveillance into ongoing MDA control programmes for trachoma, schistosomiasis and STH within Kenya to acquire substantial data on community-level incidence of SBE and associated hospital seeking rates. We also sought to assess the costs of this integrated approach for capturing SBE burden data.

## Methods

### Ethics statement

Consultative forums with community members were organized during the advocacy meetings. The study was approved by the Moi Teaching and Referral Hospital/ Moi University College of Health Sciences - Institutional Research and Ethics Committee (MTRH/MU-IREC) in Kenya (approval number FAN: 0004631). A research license was obtained from NACOSTI (NACOSTI/P/24/34290). Data was aggregated at the level of the community drug distributor, as common practice in MDA, in order to anonymise individual data from snakebite survivors.

### Study setting and design

We conducted a cross-sectional community survey nested within all campaigns targeting trachoma, schistosomiasis and STH in Kenya in the study period. We collected data through MDA campaigns in 17 of Kenya’s 47 counties. The selection of these counties was based solely on the need for the MDA campaign [14]. Data was obtained between July 2022 and May 2023 from five counties nested within the MDA for trachoma (Turkana, West Pokot, Narok, Kajiado and Baringo) and between December 2022 and August 2023 from 12 counties nested within the MDA for schistosomiasis and STH (Kakamega, Bungoma, Vihiga, Trans Nzoia, Lamu, Kilifi, Mombasa, Siaya, Kisumu, Homa Bay, Migori and Busia).

### Integration of snakebite into MDA campaigns

MDA for trachoma, schistosomiasis and STH is delivered in endemic counties in Kenya which exceed a target prevalence threshold, with the decision to deliver the campaign made at the subcounty level, based on prevalence. For trachoma, MDA is one component of the SAFE strategy initiated by the WHO, with the aim of achieving disease elimination [15]. In communities with a baseline prevalence of 5% or greater, MDA is undertaken annually for three years with a target coverage rate of 80%. Children under 6 months of age receive tetracycline eye ointment; all others receive a single dose of azithromycin [16]. For schistosomiasis and STH, a combined strategy to reduce morbidity is implemented, as the control tools are similar for both conditions [17]. MDA with praziquantel and albendazole/mebendazole is undertaken in communities with a schistosomiasis prevalence above 1% and or STH prevalence above 2%. The exercise is repeated for 3–5 years with a target coverage of 75% [11]. Upon the completion of the exercise, a mop-up exercise was conducted in areas where the initial targets had not been met.

The opportunity to obtain better data on burden by integrating snakebite questions into the MDA programmes for trachoma, schistosomiasis and STH was initially discussed with key government and non-governmental stakeholders. For each county, meetings were organized to prepare implementation and tools for the MDA programme, involving national government officials, key stakeholders, donor representatives, and the Kenyan Snakebite Research and Interventions Centre (K-SRIC) team. The K-SRIC team dedicated six hours of sessions per county to discuss the integration of snakebite questions into the MDA programme. These sessions provided a general background on snakebites, including the epidemiological relevance and importance of collecting accurate data. County NTD supervisors took the lead in training community drug distributors, sub-county and ward supervisors on data collection and reporting tools, including incorporation of snakebite incidence data into the integrated data collection tools.

Beginning approximately 2–3 weeks prior to the MDA, mobilization and advocacy for both the MDA and integration were conducted at both sub-county and community levels utilizing various communication channels to disseminate key messages. These included posters, flyers, television broadcasts, and local radio channels. Additionally, information was shared through community health unit WhatsApp groups. By employing a multi-channel approach, these efforts aimed to ensure widespread awareness and participation among community members, thereby maximizing the effectiveness of both the MDA and integration initiatives.

Throughout the duration of the MDA, stakeholders engaged in daily online meetings to receive reports, address any emerging issues, verify the attainment of daily population targets, and confirm that the health care workers and community volunteers of the local community, who were trained and equipped to distribute the MDA medications to eligible individuals, diligently administered the snakebite questions.

### Mass Drug Administration procedures

Snakebite questions were incorporated into the standard form which is used to record information on individuals receiving MDA, including age, sex, and ward/(sub)county. The schistosomiasis and STH MDA included individuals above 12 months old. The trachoma MDA included individuals without age restrictions. Age was collected in categories as relevant for the administration of drugs in the MDA interventions. For trachoma, these were < 6 months, 6 months – 7 years, and > 7 years. For schistosomiasis/STH, these were 1– 5 years, 6–14 years and > 14 years. The data was aggregated at ward level by disease-specific programme leads at the Kenyan Ministry of Health.

Community drug distributors asked every individual receiving the MDA one or two questions on snakebite: first, whether the individual had experienced a snakebite in the last 12 months and if yes, whether they sought care at a health facility. A snakebite was considered as a bite from any snake or experiencing venom in the eye(s) from a spitting snake. Response was sought from accompanying parents or guardians in the case of children unable to answer for themselves. Reports of bites were excluded if the individuals were uncertain which animal had caused the bite.

### Health facility data

Health facility data on snakebite cases were extracted in February, 2024 from the Kenya Health Information System (KHIS). The data were filtered by county and the 12 months covered by the MDA-acquired snakebite data of this study. KHIS is an integrated, national, digital platform that collects, manages, and analyses health data across Kenya, providing real-time information for health professionals and policymakers to make informed decisions.

### Statistical analysis

We summarised descriptive statistics on snakebite cases, including age (in categories collected via MDA) and sex. For snakebite burden, we presented raw snakebite case numbers, incidence rate per 100,000 person-years for each county and a weighted case count, adjusting raw case numbers for MDA coverage rates given the total target population obtained from the 2019 Kenya Census [14]. Risk ratio (RR) was calculated using the unconditional maximum likelihood estimation with a normal approximation of the confidence interval.

We also assessed correlation between sub-county level snakebite incidence and population density reported in the 2019 Census [14]. Maps showing the burden of snakebite (incidence/100,000 person-years) were generated for visualization of within-county differences. Incidence data for each county was compared to available published figures from community surveys or hospital data.

For data on hospital attendance, we presented the raw number of snakebite survivors who reported attending a health facility for snakebites in each county. Additionally, we provide the estimated weighted count, which adjusts the raw numbers to correct for MDA coverage rates.

Furthermore, we estimated the proportion of hospital attendances identified through the MDA compared with the hospital attendances reported through KHIS. This comparison helps to evaluate the consistency and coverage of reporting between these two data sources, providing insights into the reliability and completeness of the data.

Data analysis was performed using R software version 4.3.0 (R Core Team, 2023). Maps were produced using QGIS Geographic Information System version 3.40 (QGIS Development Team (2024). Administrative boundary shapefiles were obtained from GADM database of Global Administrative Areas (version 4.1, 2022), used under license for academic and non-commercial purposes. All maps were generated from spatially processed data in the EPSG:4326 WGS 84 coordinate reference standard.

## Results

A total of 13,117,754 persons from 17 counties were included in the MDA surveys, representing 27.6% of Kenya’s total population. 687,2155 (52.4%) respondents were female. The percentage of females in the sampled populations ranged from 48% to 59% (median 52%). The sampled county population size varied from 132,316–1,682,003 persons with a median coverage of 93% (min. 63%, max. 112%) of the total sub-county population as recorded in the 2019 census (Table 1). Two counties, Kajiado and Lamu, did not achieve the target coverage rate of 80%, achieving instead a coverage rate of 63% and 75% respectively. A second MDA intervention was organized in these counties but did not include the questions on snakebite for logistic reasons.

**Table 1:** Mass Drug Administration (MDA) Coverage per county.

County	Sub-counties sampled	Population target	Population sampled	Coverage
Baringo	2/7	174,043	160,625	92%
Bungoma	10/10	1,583,760	1,421,460	90%
Busia	7/7	799,011	730,246	91%
Homa Bay	8/8	1,209,330	1,359,911	112%
Kajiado	2/5	378,227	239,839	63%
Kakamega	12/12	1,696,658	1,682,003	99%
Kilifi	7/7	1,408,041	1,293,914	92%
Kisumu	7/7	845,697	749,678	89%
Lamu	2/2	177,539	132,316	75%
Migori	7/8	1,035,433	1,003,743	97%
Mombasa	6/6	981,112	896,200	91%
Narok	3/6	587,844	507,374	86%
Siaya	6/6	886,285	879,254	99%
Trans Nzoia	5/5	956,142	790,489	83%
Turkana	4/7	491,689	417,548	85%
Vihiga	5/5	607,575	615,279	101%
West Pokot	2/4	271,398	237,875	88%

### Snakebite incidence

A total of 4,667 cases was reported across the 17 counties; 52.6% male. Males had a slightly higher incidence rate of 39.3 cases per 100,000 inhabitants (95% CI 37.8-40.1), compared to females (32.2 cases per 100,000 inhabitants, [95% CI 30.9-33.6]). Females had an 18% lower chance of having a snakebite (RR = 0.82, 95% CI = 0.77-0.87).

For all the (12) counties where schistosomiasis and STH MDA was conducted, snakebite cases were divided into three age groups (1–4 years, 5–14 years, and 15+). Of the total 1,655 cases, 108 snakebite survivors were aged 1–4, 352 were aged 5–14 and 1,195 were aged 15 or above. The lowest snakebite incidence rate was reported in children 1–4 years old at 5.6 cases per 100,000 inhabitants (95% CI 4.6-6.8). Children aged 5–14 reported a snakebite incidence rate of 9.2 (95%CI 8.3-10.2) cases per 100,000 inhabitants and those aged 15 and above had a rate of 19.5 cases per 100,000 inhabitants (95%CI 18.4-20.7).

**Fig 1:**
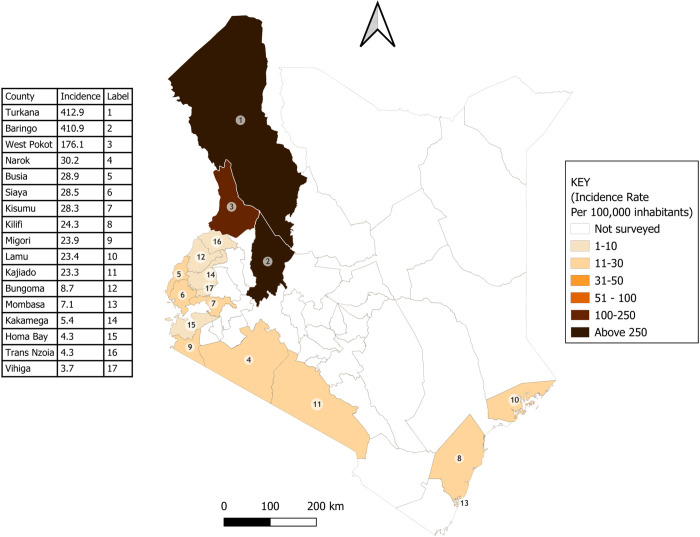
Between county variation in snakebite incidence in Kenya. Base map: GADM database of Global Administrative Areas, version 4.1 (www.gadm.org). Used under license for academic, non-commercial use (https://gadm.org/license.html). Maps created in QGIS version 3.40 (QGIS Development Team. QGIS Geographic Information System. Open-Source Geospatial Foundation Project. http://qgis.osgeo.org).

The overall incidence rate of snakebite in all the counties surveyed was 35.6 (95% CI 34.6 – 36.6) cases per 100,000 inhabitants/year at risk. Incidence rates varied greatly between the counties (Fig 1). Turkana County had the highest incidence of 412.9 snakebites per 100,000 inhabitants/year (95% CI 393.6 – 432.8), closely followed by Baringo County (410.9 [95% CI 380.1-443.5]). Vihiga County recorded the lowest incidence rate of 3.7 snakebites per 100,000 inhabitants/year (95% CI 2.2-5.3-4.7). There was a very weak negative correlation (rho -0.14) between subcounty level incidence rates of snakebite and the population density.

Table 2 presents the snakebite counts and incidence by county. Inverse weighting was applied based on MDA coverage to provide estimated total case numbers for the target population in each county if 100% coverage was not met.

**Table 2:** Population density and snakebite incidence per county, as reported by community members over the past twelve months.

COUNTY	Population density (people/km^2^)	Total cases reported	Weighted cases	Incidence rate (cases/100,000 people/year (95% CI))	Lowest sub-county incidence rate	Highest sub-county incidence rate
Baringo	61	660	715	410.9 (380.1-443.5)	410.9	410.9
Bungoma	552	124	138	8.7 (7.3-10.4)	0.7	36.9
Busia	527	211	231	28.9 (25.1-33.1)	2.5	152.9
Homa Bay	359	59	52	4.3 (3.3-5.6)	0	25.6
Kajiado	51	56	88	23.4 (17.6-30.3)	18.7	27.8
Kakamega	618	91	92	5.4 (4.4-6.6)	0	18.9
Kilifi	116	315	343	24.3 (21.7-27.2)	3.8	59.8
Kisumu	554	212	239	28.3 (24.6-32.4)	2.0	61.3
Lamu	23	31	42	23.4 (15.9-33.3)	13.7	24.6
Migori	427	240	248	23.9 (21.0-27.1)	0	100.5
Mombasa	5495	64	70	7.1 (5.5-9.1)	0	29.9
Narok	65	153	177	30.2 (25.6-35.3)	18.4	44.5
Siaya	393	251	253	28.6 (25.1-32.3)	14.1	71.8
Trans Nzoia	397	34	41	4.3 (2.8-5.7)	2.1	9.4
Turkana	14	1724	2030	412.9 (393.6-432.8)	169.5	841.4
Vihiga	1047	23	23	3.7 (2.2-5.3)	0	5.1
West Pokot	68	419	478	176.1 (159.7-193.8)	165.3	188.7

**Fig 2:**
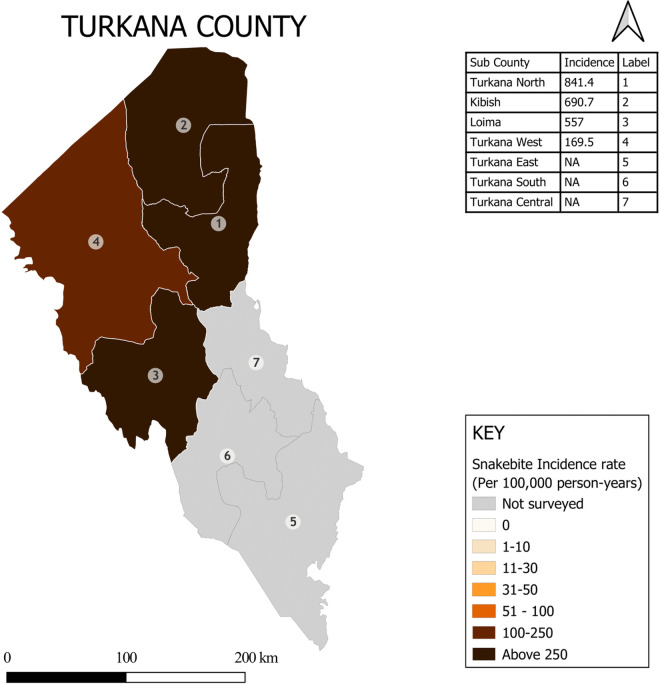
Within county variation in snakebite incidence in Turkana County (A high incidence setting). Base map: GADM database of Global Administrative Areas, version 4.1 (www.gadm.org). Used under license for academic, non-commercial use (https://gadm.org/license.html). Maps created in QGIS version 3.40 (QGIS Development Team. QGIS Geographic Information System. Open-Source Geospatial Foundation Project. http://qgis.osgeo.org).

**Fig 3:**
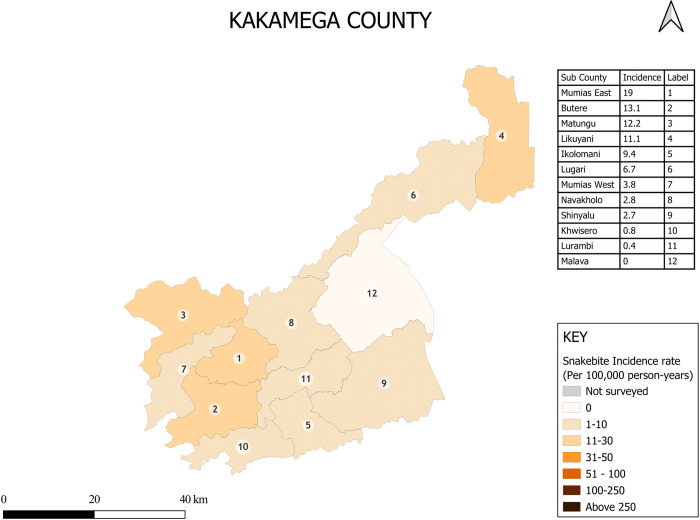
Within county variation in snakebite incidence in Kakamega County (A low incidence setting). Base map: GADM database of Global Administrative Areas, version 4.1 (www.gadm.org). Used under license for academic, non-commercial use (https://gadm.org/license.html). Maps created in QGIS version 3.40 (QGIS Development Team. QGIS Geographic Information System. Open-Source Geospatial Foundation Project. http://qgis.osgeo.org).

Snakebite incidence varied less within counties than between counties. The maps of Turkana County and Kakamega County show examples of within-county variation in snakebite incidence in high and low incidence settings respectively (Figs 2 and 3). Detailed maps presenting sub-county and county level incidence rates for all 17 counties are provided in S1–S15 Files.

### Comparison with previous studies

The data presented in Table 3 below illustrates how our study, which captured community-level incidence of snakebite through MDA integration, provides significantly higher estimates of incidence in several counties compared to prior published hospital-based studies and household level surveys. For example, in Baringo County, comparing our community-based estimate of 410.9 cases per 100,000 people per year with the hospital-based studies (e.g., 67.9/100,000 by Coombs et al. and 6.7/100,000 by Ochola et al.). In contrast, our lower incidence estimates in counties such as Kakamega and Busia are consistent with previous findings.

**Table 3:** Snakebite incidence rate in counties covered in this study compared to incidence in published literature.

STUDY DETAILS	SBE-MDA Integration (This study)	Snake bites in Kenya: a preliminary survey of four areas [9]	Epidemiology of snake bites in selected areas of Kenya [18]	A long-term observational study of paediatric snakebite in Kilifi County [19]	The Burden of Snakebite in Rural Communities in Kenya [20]	Snakebite victim profiles and treatment-seeking behaviours in Kenya [21]
**STUDY METHOD**	Community-level surveys	Retrospective study design using hospital records	Retrospective study design using hospital records	Hospital-based prospective study design	Household-level survey	Household-level survey
**INCLUSION CRITERIA**	All individuals residing in the study area	Sample of 50 selected health facilities	All patients presenting to Kakamega provincial hospital, Kabarnet, Kapenguria and Makueni district hospitals	All children presenting to Kilifi County Hospital	A random sample of 93 respondents in Kaloleni Sub County, Kilifi	All households within Mbita Sub County, Homa Bay
**SBE INCLUSION PERIOD**	2021/2022	1990-1995	2007-2009	2003-2021	2019	2011-2016
**INCIDENCE RATES**	**Cases/100,000 people/year (95% CI)**				**Lifetime risk of bite (%)**	**Households reporting at least 1 snakebite (per 10,000 HHs/year)**
**BARINGO**	410.9 (379.6-442.2)	67.9	6.7			
**BUSIA**	28.9 (25.0-32.8)	25.3				
**HOMA BAY**	4.3 (3.2-5.5)					100.3
**KAJIADO**	23.35 (17.2-29.5)					
**KAKAMEGA**	5.41 (4.3-6.5)	1.9	4.6			
**WEST POKOT**	176.1 (159.7-193.8)		2.7			
**KILIFI**	24.34 (21.6-27.0)	44.0		11.3 (0–5 years) 29.1 (6–12 years)	14% (based on 93 respondents)	

### Health facility attendance following snakebite

In the 12 counties where SCH/STH MDA was conducted, health facility seeking rate was disaggregated by gender and age groups. There was no notable difference between the health facility seeking rate after a snakebite between the male (63%, 554 of 882) and female (67%, 520 of 773) population in these counties. Following a snakebite, the health facility seeking rate was 51% (n = 55) for children aged 1–4.The rate was 67% (n = 236) in those aged 5–14, and 65% (n = 783) in individuals aged 15 and older). The proportion of snakebite survivors reporting having sought health facility care varied across all the 17 counties, from 3% in Trans Nzoia County to 86% in Kajiado County (median overall 66%). Aside from West Pokot, in all counties the number of snakebite survivors reported having attended a health facility were lower than the data reported in the Kenya Health Information System (KHIS) for the same region and period (Table 4). Table 4 compares the number of health facility visits reported for snakebite in the MDA surveys to snakebite case numbers reported through the KHIS.

**Table 4:** Comparison between snakebite related health facility (HF) visits based on the MDA exercise and data reported in the KHIS data. Weighting was done at the sub county level. *Percentages <100 indicate lower number of hospital visits as reported in the communities compared to numbers in HFs.

County	MDA				KHIS	MDA HF reports as % of KHIS reports*
	Weighted cases of snakebites in county	Cases reporting HF visit in county	Weighted HF visits in county	% cases of snakebites reporting HF visit	Snakebite cases reported in hospital data	
Baringo	715	433	469	66%	486	97%
Bungoma	138	11	12	9%	613	2%
Busia	231	168	184	80%	549	34%
Homa Bay	52	35	31	59%	320	10%
Kajiado	88	48	76	86%	287	26%
Kakamega	92	35	35	38%	439	8%
Kilifi	343	224	244	71%	1042	23%
Kisumu	239	160	180	75%	297	61%
Lamu	42	18	24	58%	64	38%
Migori	248	165	170	69%	352	48%
Mombasa	70	42	46	66%	91	51%
Narok	177	95	110	62%	139	79%
Siaya	253	203	205	81%	280	73%
Trans Nzoia	41	1	1	3%	109	1%
Turkana	2030	1296	1526	75%	1835	83%
Vihiga	23	12	12	52%	128	9%
West Pokot	478	261	298	62%	119	250%

### Costs of the integrated approach

The integration of snakebite data collection into this framework incurred only an extra £2,802. The costs for this integration were limited to stipends and in-country travel. This includes KSRIC team members responsible for training MDA staff on the rationale for including snakebite envenoming in the programme and guiding them on how to collect the information during the MDA exercise. In comparison, a standalone sample survey on snakebites in Turkana County run by LSTM and KSRIC targeted 10,494 individuals (1789 households) required £24,118 and over 3 months of operations, to cover all staffing, materials, community sensitization and logistical costs. The cumulative cost of conducting the MDA exercise itself ranged between £30,000 and 78,000 per subcounty depending on the sub county’s population. Using Turkana County as a representation of participating counties, the cost of running the MDA exercise amounted to an estimated £120,000.00 for one month of operations, which reached a target of 417,548 participants.

## Discussion

A lack of geographic and demographic data on snakebite envenoming incidence within countries has been noted as a barrier to effective distribution of antivenom [22,23]. Our collaboration with NTD MDA campaigns has provided the most extensive data on community snakebite incidence in Kenya to date. Between January 2020 and August 2023, we obtained high quality data from 17 counties, accounting for 27.6% of the country’s population. The work uniquely captures the considerable variation in incidence rates across an endemic country, and in doing so increases our knowledge of incidence in both high and relatively lower burden regions; the latter are often overlooked as studies tend to focus on areas where snakebite envenoming is a known risk [22,24,25]. Incorporating snakebite data capture within the MDA campaigns has provided rich epidemiological data from which policy makers can effectively plan resource allocation for snakebite treatment and prevention.

We found an overall snakebite incidence of 36 cases/100,000 people/year, with figures as high as 413/100,00/year in Turkana, 411/100,000/year in Baringo and 176/100,000/year in West Pokot [9,18,20,26–30].

The data we have obtained from 17 of the 47 Kenyan counties to date shows a clear geographical pattern, with the highest estimated incidence rates clustered in counties to the northwest. We found a weak negative correlation between county-level incidence and population density and within counties (between sub-counties). Snakebite risk generally tends to be higher in sparsely populated rural areas [31–34]. That our study shows a weak correlation likely reflects the multiple factors accounting for the risks at subcounty level. Population density is just one of several spatially varying factors accounting for the geographical variation in snakebite risk, with climate and environmental factors affecting snake habitat suitability [26], and sociodemographic factors affecting human-snake interaction [32,35,36]. Prevention and control efforts should reach remote rural populations with typically the poorest access to healthcare services [21,37]. In future work, conducting geospatial modelling would allow us to further utilise this data, to model and predict risk across a larger area.

Within the MDA campaigns, we collected data on the proportion of snakebite survivors who reported attending a health facility. Our results show considerable variation in the proportion reporting healthcare attendance across the counties, ranging from as low as 3% in Trans Nzoia to 86% in Kajiado. There are many reasons documented for why patients might not seek care in formal health facilities, including long travel distances, high cost of treatment compared to traditional or self-treatment and local traditions and beliefs [20,38], however we did not observe a correlation between healthcare attendance and population density. Due to the limited number of questions we were able to ask, it was not possible for us to capture detail on the reason for patients not seeking health facility care. With attendance below 70% in 11/17 counties, there is potential benefit in working with communities in these areas in order to find ways to improve healthcare access and use.

There were discrepancies when comparing the number of cases reporting health facility attendance compared to the number of snakebite cases reported by the KHIS for the same period, with estimates for 12/17 counties below 70% of KHIS figures and a range from 0.9% of the KHIS figures in Trans Nzoia to 250% in West Pokot. We expected some variation between these figures, with our estimates weighted for the county population to account for incomplete MDA coverage rates, however the degree of concordance varies substantially across counties. It is possible that KHIS figures reflect multiple reporting of cases, as patients may initially seek care at a local primary healthcare facility and are subsequently referred to higher tier facilities for antivenom administration or further management [39], or patients may be re-recorded as a snakebite case on re-attendance for follow-up care. It is also possible that the KHIS figures for one location capture patients who have travelled out of or been referred from their home locality to receive appropriate treatment. The implication of this is twofold. Firstly, standardising the collection and reporting of snakebite case information by healthcare facilities has the potential to considerably improve the utility and reliability of this routine data source for understanding snakebite burden across the country. Secondly, given patients may not seek care at the facility in their sub-county of residence, both survey data and KHIS reports should be considered in antivenom stock planning.

There is limited snakebite data available from Kenya, but our estimates suggest higher rates than those identified from previous hospital-based studies [18]. Incidence estimates based on hospital data are known to underestimate true figures, as many snakebite patients, particularly in remote rural locations, seek treatment from traditional healers rather than using the formal healthcare system, whilst those developing complications from severe envenoming may die before they reach a health facility [2,20,26]. Notably, there was a discrepancy between the counties reporting the highest number of cases through the MDA surveys and the KHIS, with only two of the top five counties by MDA data (Turkana and Kilifi) in the top five for KHIS reports. In this context, our data provides an important addition to the literature from community based-studies which seeks to provide a more complete reflection of the snakebite burden across Kenya. There is relatively little high quality snakebite incidence data available more broadly from sub-Saharan Africa with which to compare our findings. A review of the global burden of snakebite estimated an upper incidence for the East Africa region of 22.61/100,000 [2], though it notes the paucity of data available to base this on. Elsewhere, the majority of data comes from small community studies conducted in the West and Central Africa regions and our highest incidence rates fall within the middle to upper range of these figures, similar to those found in studies from Cabo Delgado, Mozambique (352/100,000) [27] and Benue Valley, Nigeria (497/100,000) [28] but lower than very high rates found in areas including Cameroon (665/100,000) [29], and Bandafassi, Senegal (915/100,000) [40].

Our study successfully integrated two key questions on snakebite burden across several large MDA surveys, allowing us to rapidly obtain information on this condition for a quarter of Kenya’s population for less than £3000 in addition to existing costs for the MDA [6,41]. A systematic review identified integrated NTD strategies to be cost effective [42]. In line with the WHO’s call for greater integration across NTD programmes, to ‘improve [their] cost-effectiveness, coverage and geographical reach’ [41], this study highlights the considerable benefits from collaboration across NTD programmes. Integration often combines activities in health education, management or control. Our study illustrates how combining different types of activities can be beneficial; elimination efforts for established NTDs with data collection on basic epidemiology for the more recently recognized NTD snakebite envenoming. As an NTD with very little formally published data [2], such approaches have the potential to provide significant benefits for understanding snakebite epidemiology across high-burden countries.

This study has limitations that should be considered. Firstly, there are factors which may have impacted the accurate reporting of snakebite. It is possible that recall bias may have led to snakebite cases being misclassified either within or outside of the one-year time frame of interest. Where this has occurred, it is likely to have affected data collection similarly across counties and therefore the variation between counties is unlikely to have been affected. It is also possible that the cultural perceptions of snakebite may have affected how forthcoming some participants were in providing information; social stigma associated with snakebite [43] could have led some respondents not to disclose their experience, particularly if data collection was conducted in a public setting. Conversely, in settings in which community engagement on healthcare matters is strong, due to regular NGO presence or research activity, residents may have been more inclined to report on healthcare matters. Secondly, working within the confines of existing NTD programmes meant that data collection was limited to areas in which MDA activities were planned, which did not always cover the whole county. Finally, while this approach to data collection has provided important information on snakebite risk, the nature of integrating with existing programmes meant that the range of information we were able to gather about cases was limited and could not include (i) discriminating bites by venomous vs non-venomous snakes and (ii) data on the burden of mortality from snakebite. To further inform prevention and control strategies, gathering detailed information from priority counties on the circumstances surrounding snakebite episodes, the management received and outcomes from the bite will be important.

The data collected has enabled us to identify those areas that should be prioritised for snakebite prevention and control, and integrating with MDA programmes that are planned over the upcoming years will provide an opportunity across selected counties to both fill the gaps in data collection and repeat assessments in order to better understand the dynamics of snakebite burden over time.

## Conclusion

Our collaboration with NTD MDA campaigns has yielded comprehensive data on snakebite incidence in Kenya, revealing significant nationwide variation and offering vital insights into both high and lower snakebite-risk regions. This informs resource allocation for treatment and prevention. Integrating with MDA programmes that are planned over the upcoming years will provide an opportunity across selected counties to both fill the gaps in data collection and repeat assessments in order to better understand the dynamics of snakebite burden over time. Integrated approaches, capitalizing on existing platforms for multiple NTDs, demonstrate significant cost savings while aligning perfectly with the WHO roadmap for NTD control and elimination.

## Supporting information

S1 File Snakebite Incidence in Baringo County. (TIF)

S2 File Snakebite Incidence in Bungoma County. (TIF)

S3 File Snakebite Incidence in Busia County. (TIF)

S4 File Snakebite Incidence in Homa Bay County. (TIF)

S5 File Snakebite Incidence in Kajiado County. (TIF)

S6 File Snakebite Incidence in Kilifi County. (TIF)

S7 File Snakebite Incidence in Kisumu County. (TIF)

S8 File Snakebite Incidence in Lamu County. (TIF)

S9 File Snakebite Incidence in Migori County. (TIF)

S10 File Snakebite Incidence in Mombasa County. (TIF)

S11 File Snakebite Incidence in Narok County. (TIF)

S12 File Snakebite Incidence in Siaya County. (TIF)

S13 File Snakebite Incidence in Trans Nzoia County. (TIF)

S14 File Snakebite Incidence in Vihiga County. (TIF)

S15 File Snakebite Incidence in West Pokot County. (TIF)
