# Supplementary figures and images for: Nationwide variation of snakebite incidence in Kenya: Community surveys as an integrated NTD approach
Source: PLoS Negl Trop Dis. 2025 Nov 21;19(11):e0013732. doi: 10.1371/journal.pntd.0013732 (PMC12654943; doi:10.1371/journal.pntd.0013732)

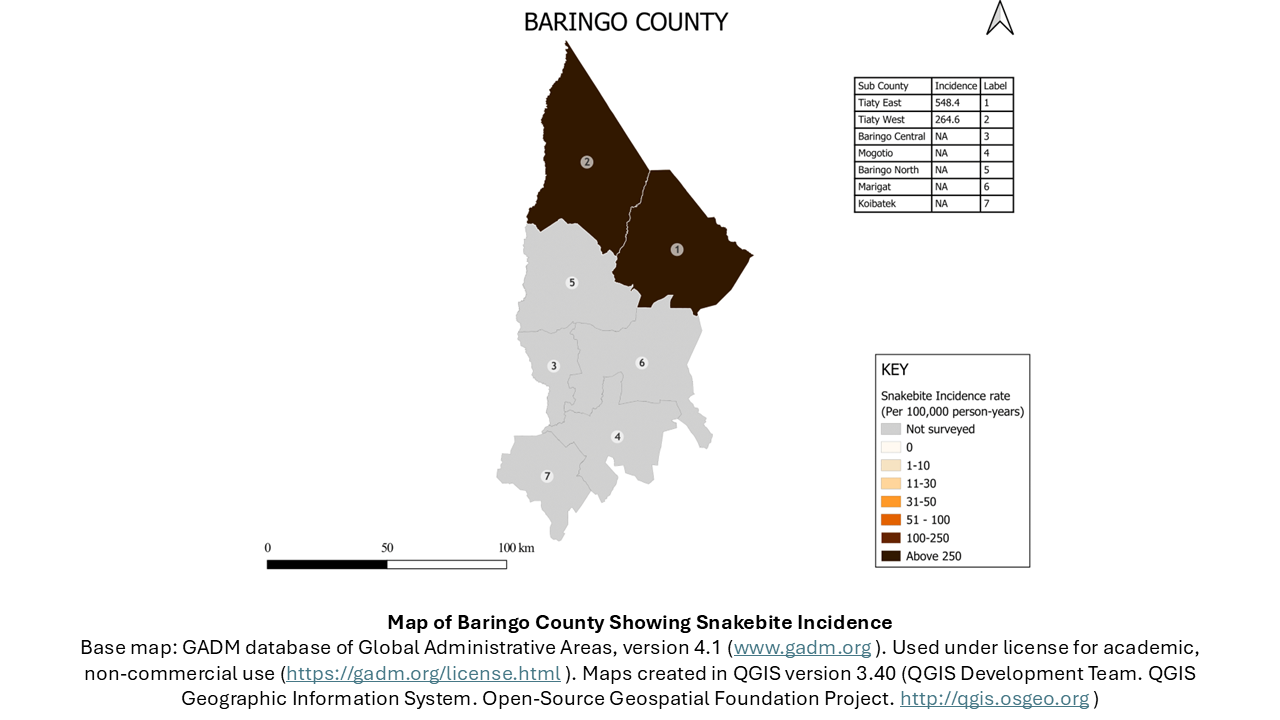

Supplement: S1 File — (TIF) [file pntd.0013732.s001.tif]

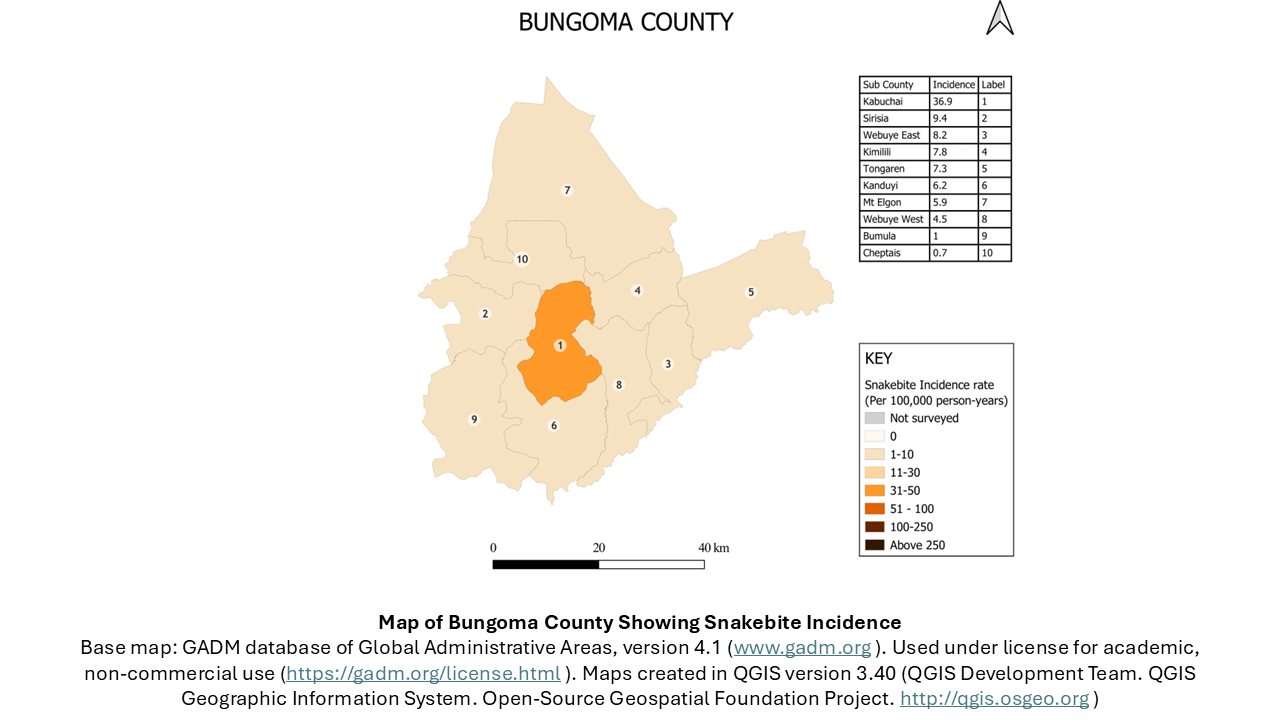

Supplement: S2 File — (TIF) [file pntd.0013732.s002.tif]

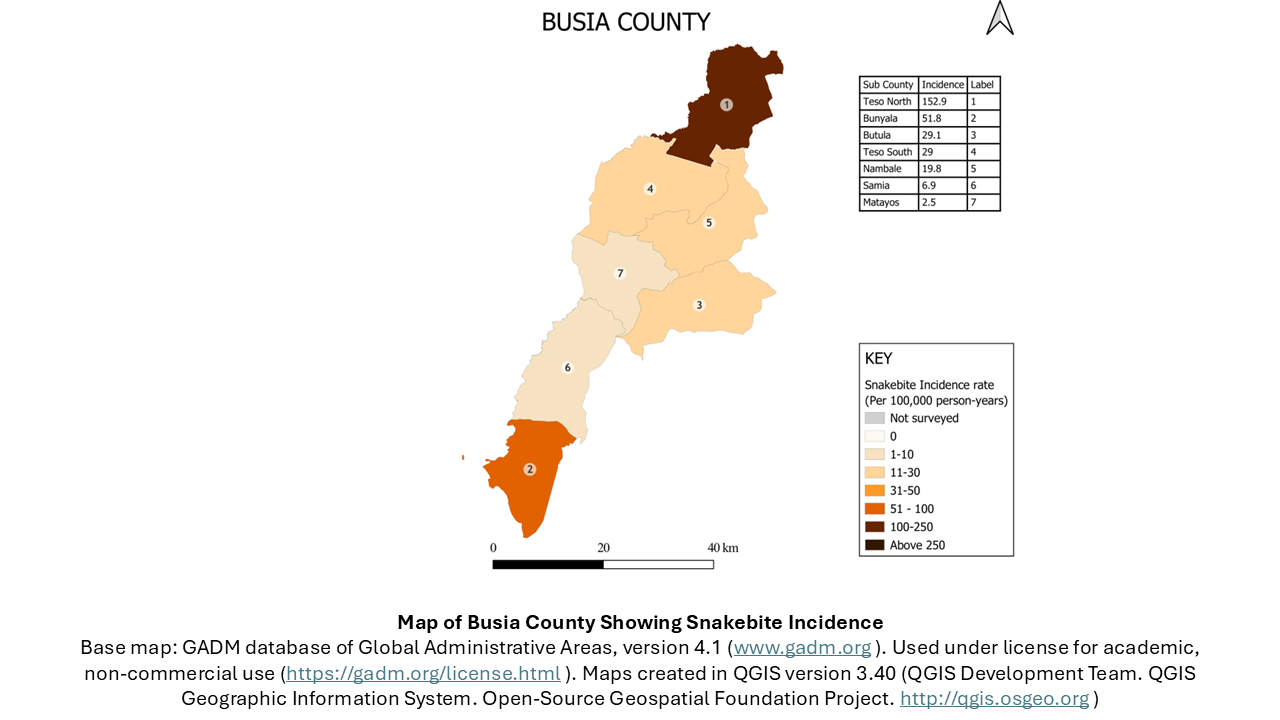

Supplement: S3 File — (TIF) [file pntd.0013732.s003.tif]

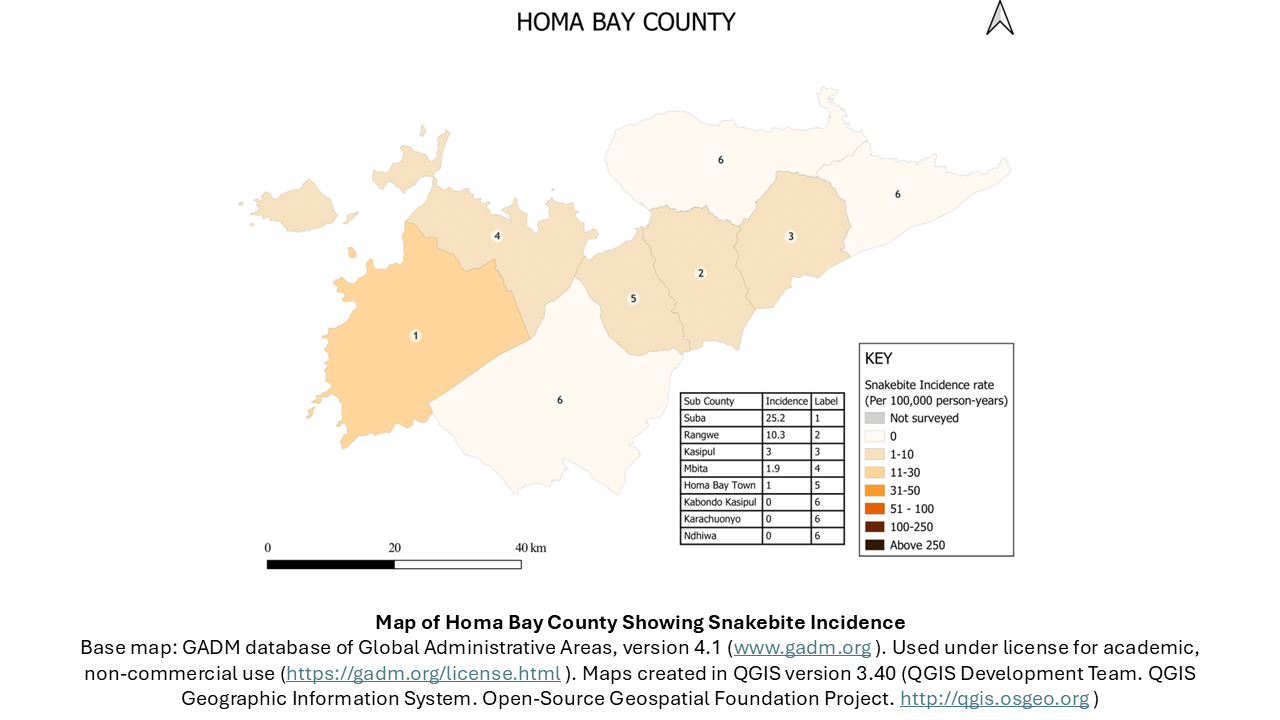

Supplement: S4 File — (TIF) [file pntd.0013732.s004.tif]

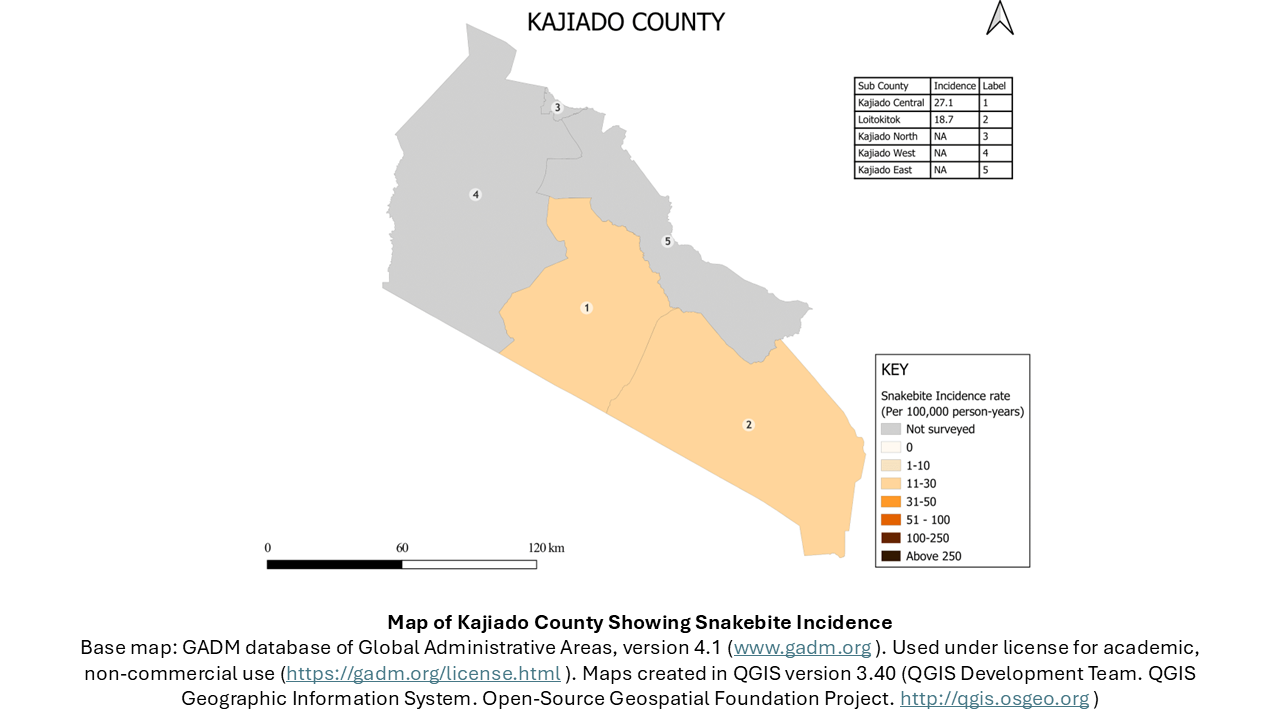

Supplement: S5 File — (TIF) [file pntd.0013732.s005.tif]

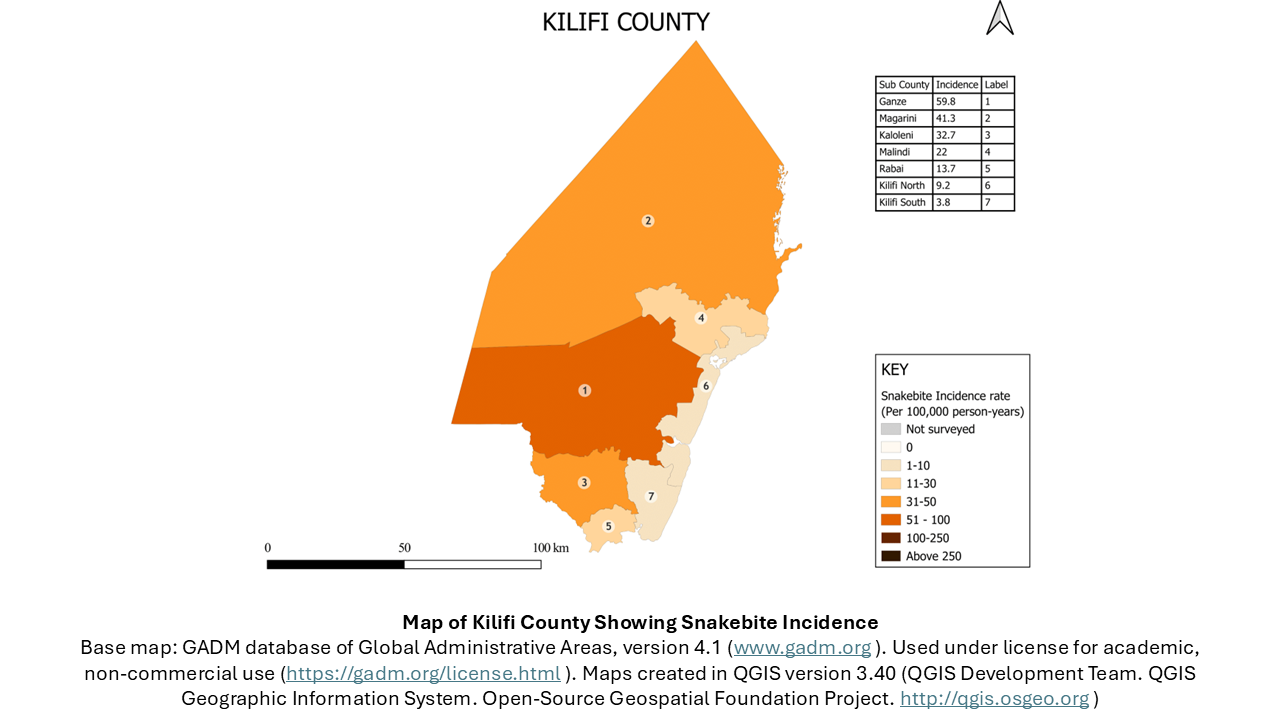

Supplement: S6 File — (TIF) [file pntd.0013732.s006.tif]

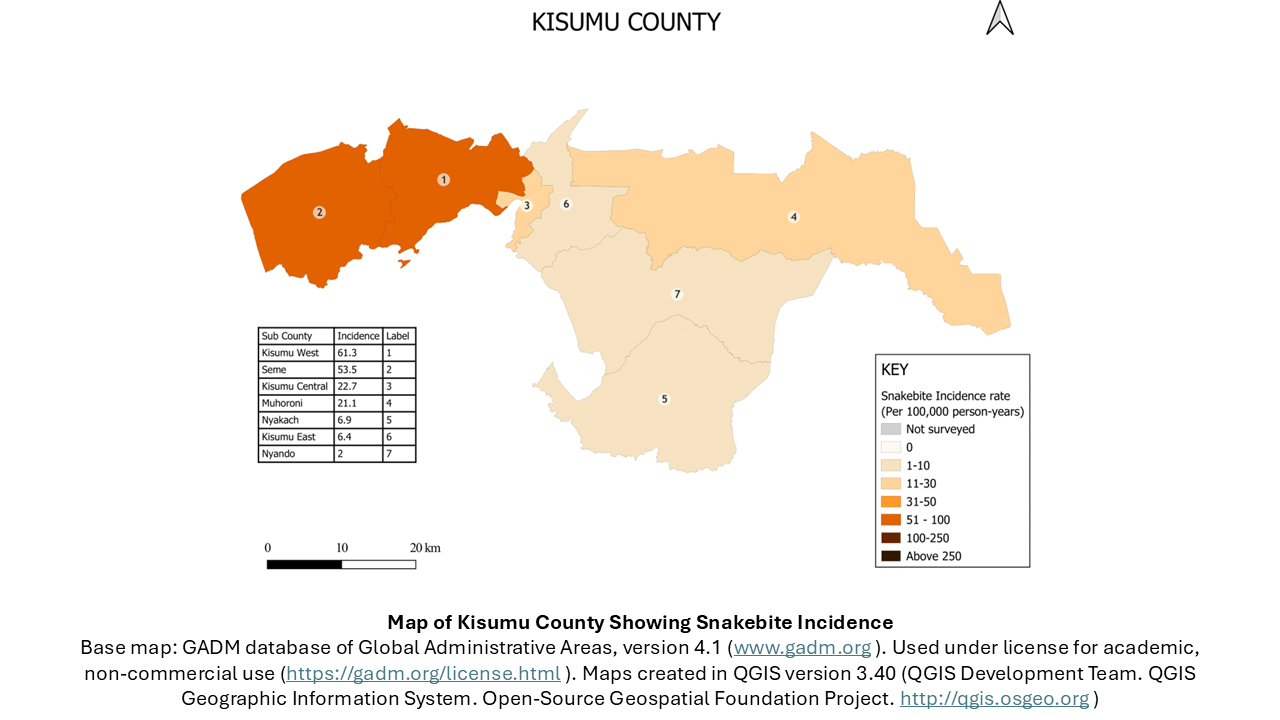

Supplement: S7 File — (TIF) [file pntd.0013732.s007.tif]

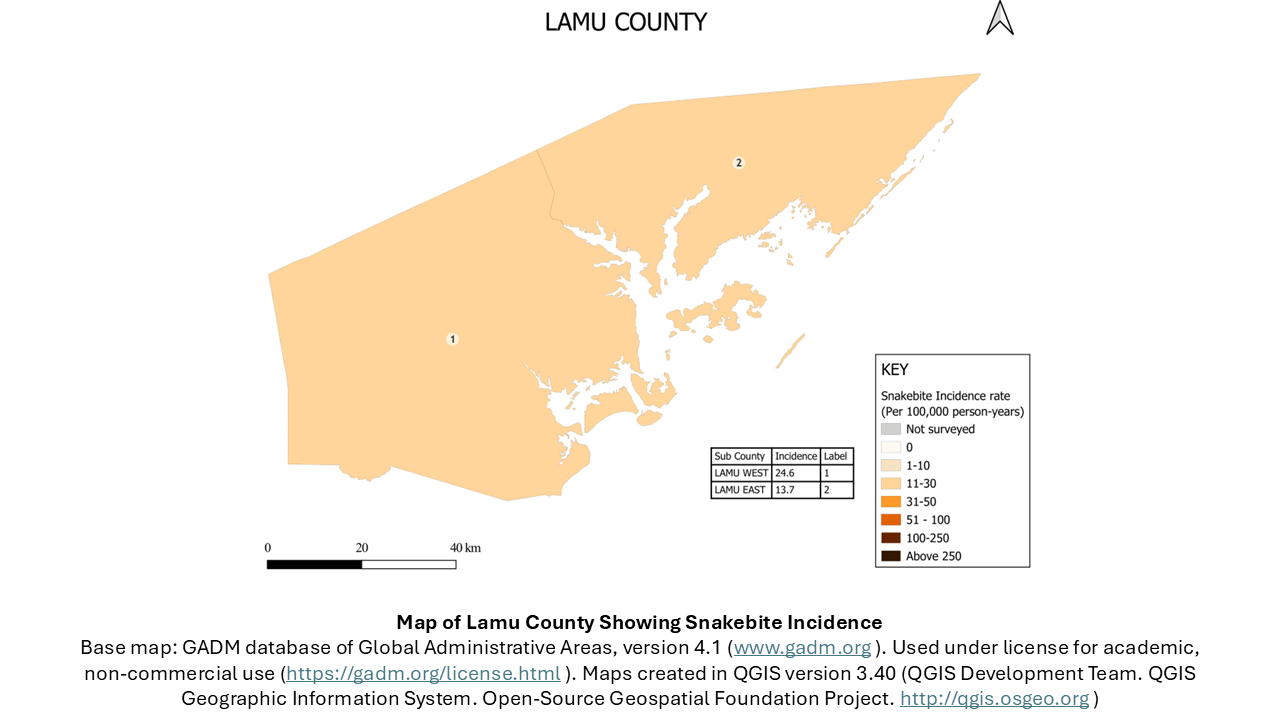

Supplement: S8 File — (TIF) [file pntd.0013732.s008.tif]

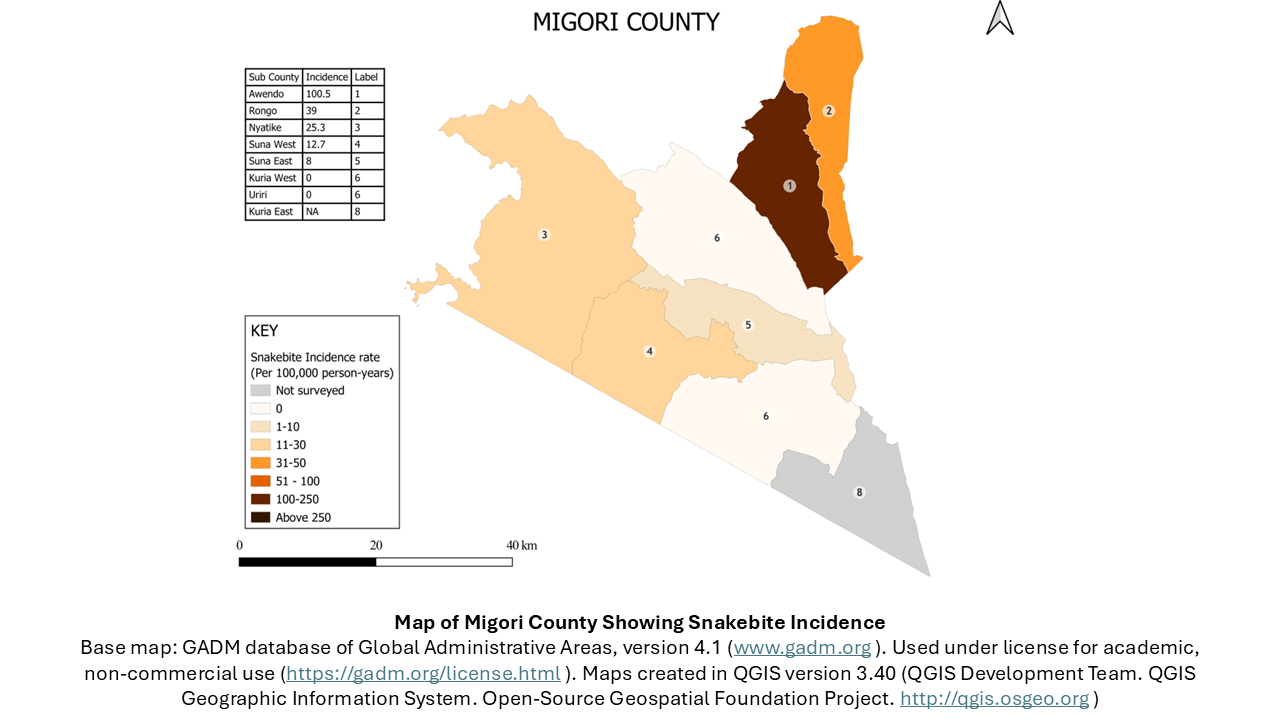

Supplement: S9 File — (TIF) [file pntd.0013732.s009.tif]

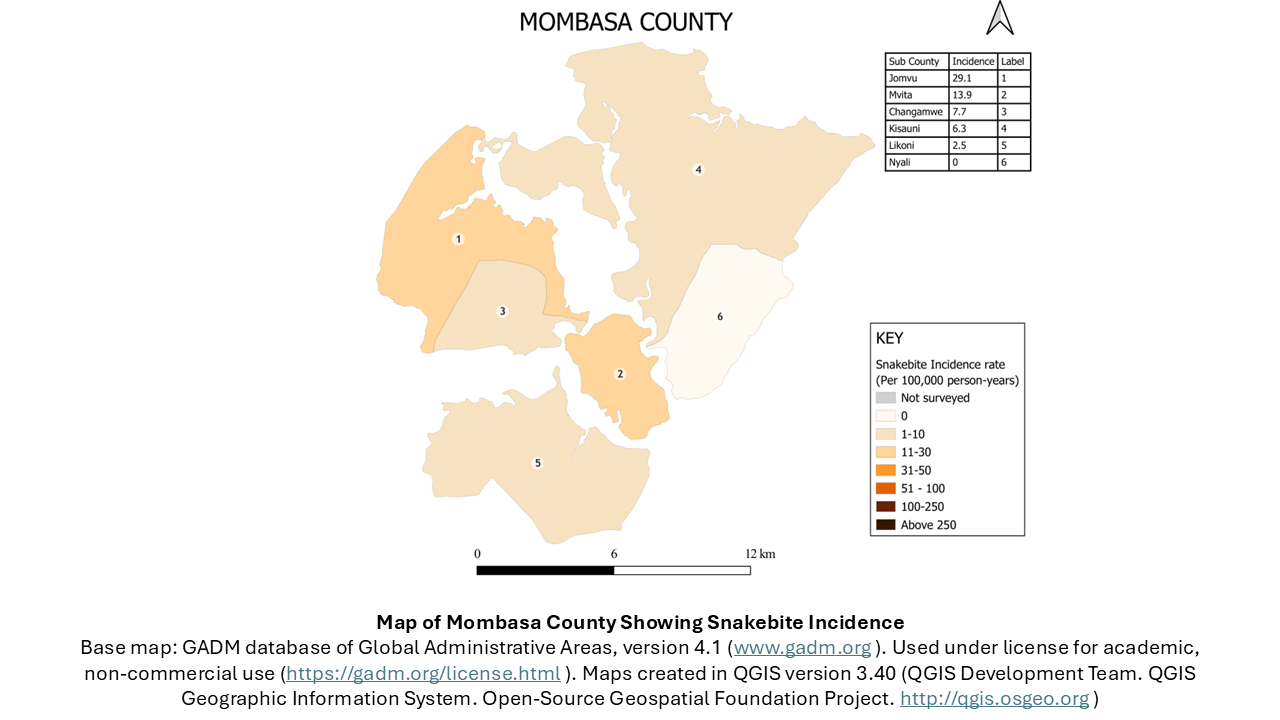

Supplement: S10 File — (TIF) [file pntd.0013732.s010.tif]

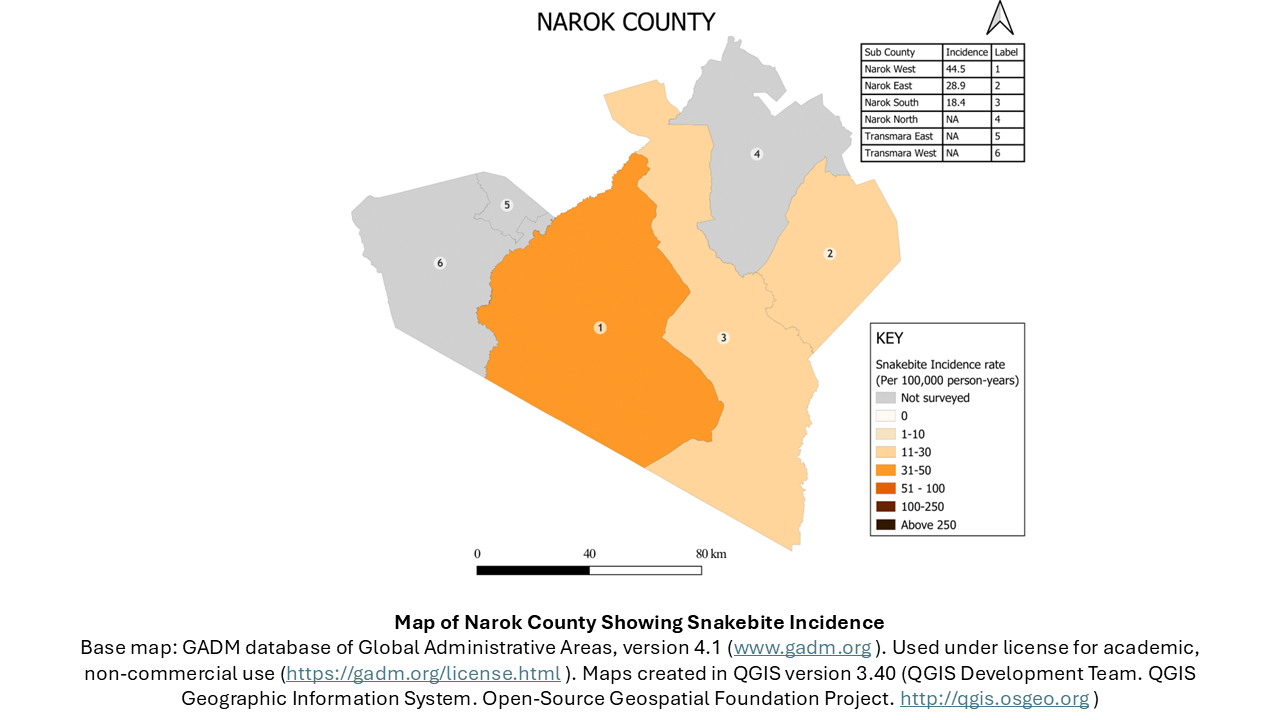

Supplement: S11 File — (TIF) [file pntd.0013732.s011.tif]

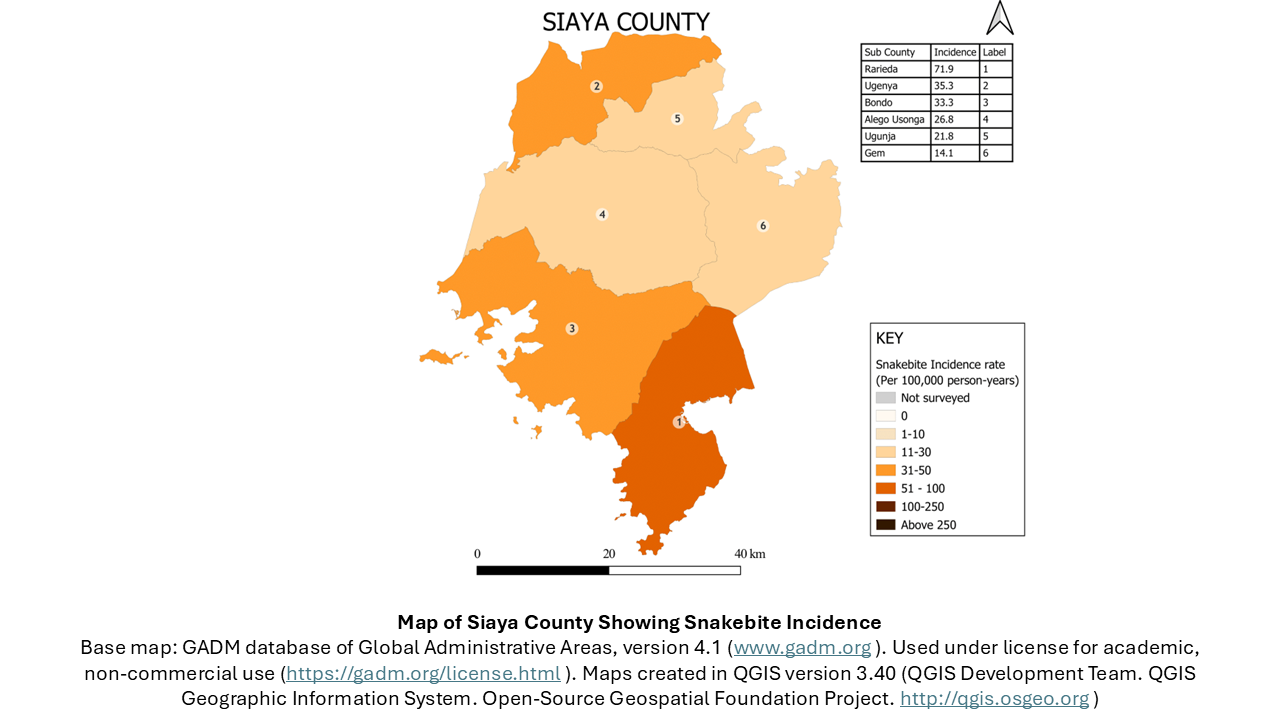

Supplement: S12 File — (TIF) [file pntd.0013732.s012.tif]

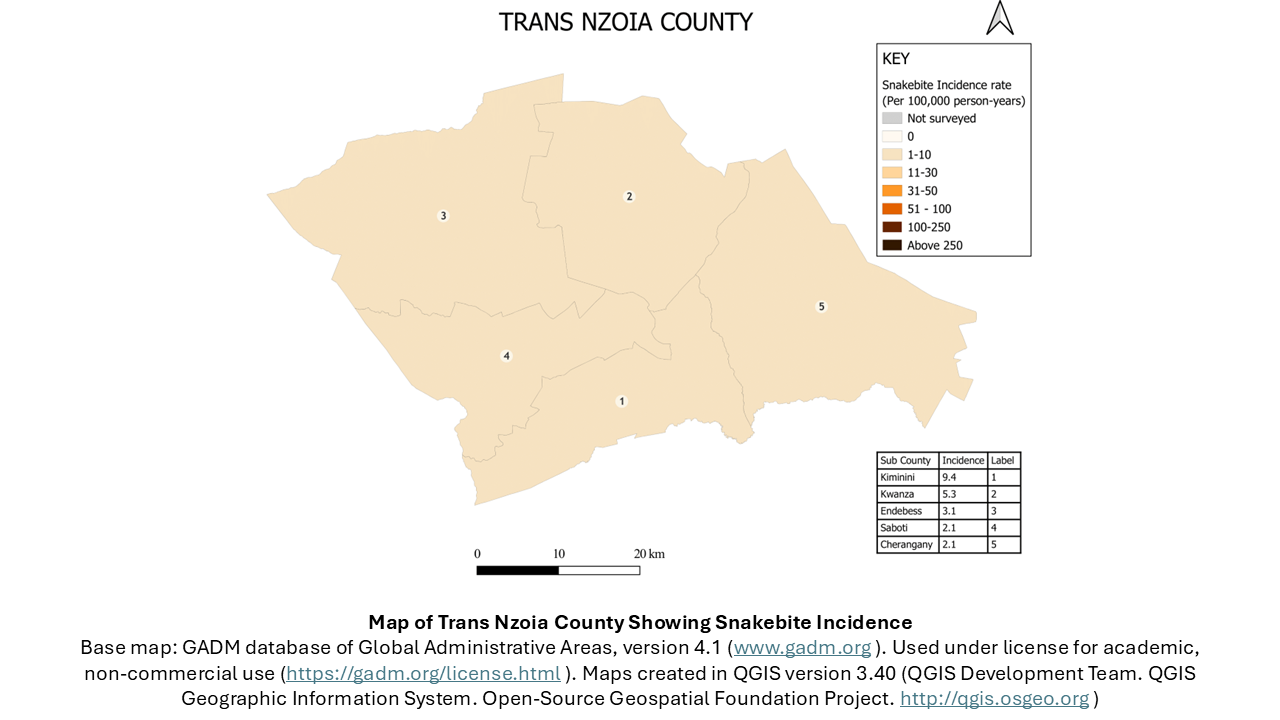

Supplement: S13 File — (TIF) [file pntd.0013732.s013.tif]

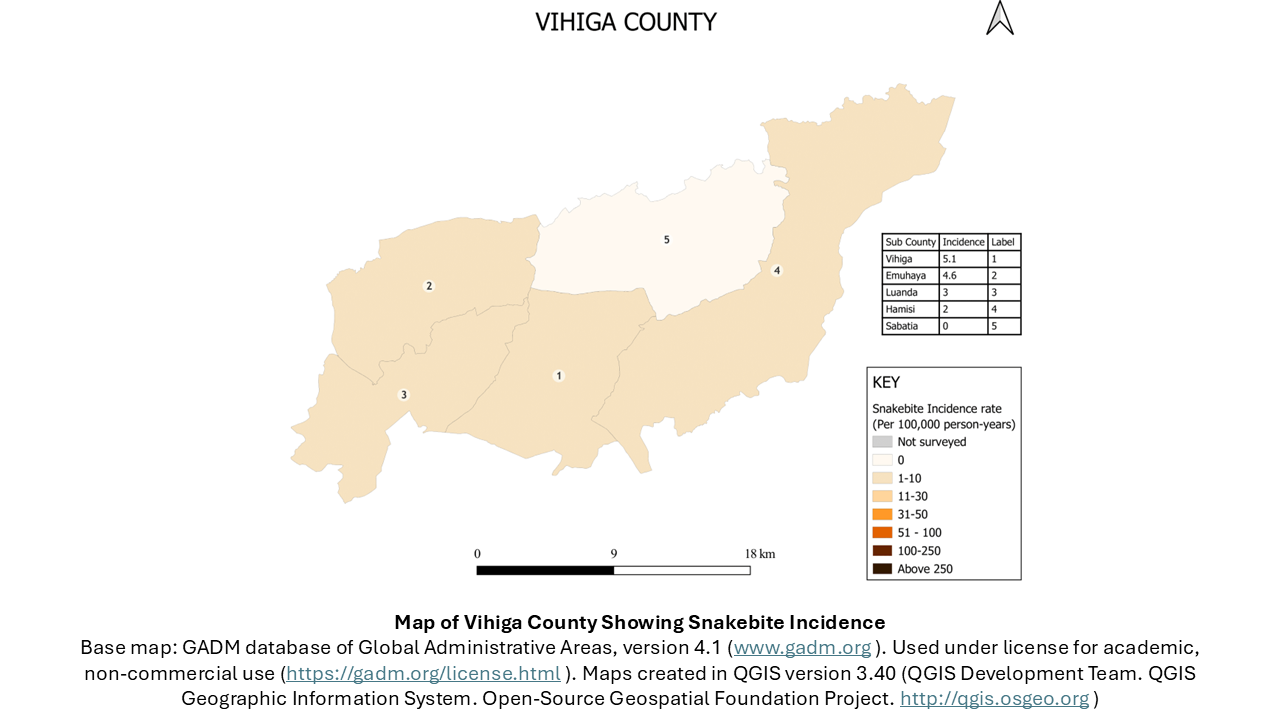

Supplement: S14 File — (TIF) [file pntd.0013732.s014.tif]

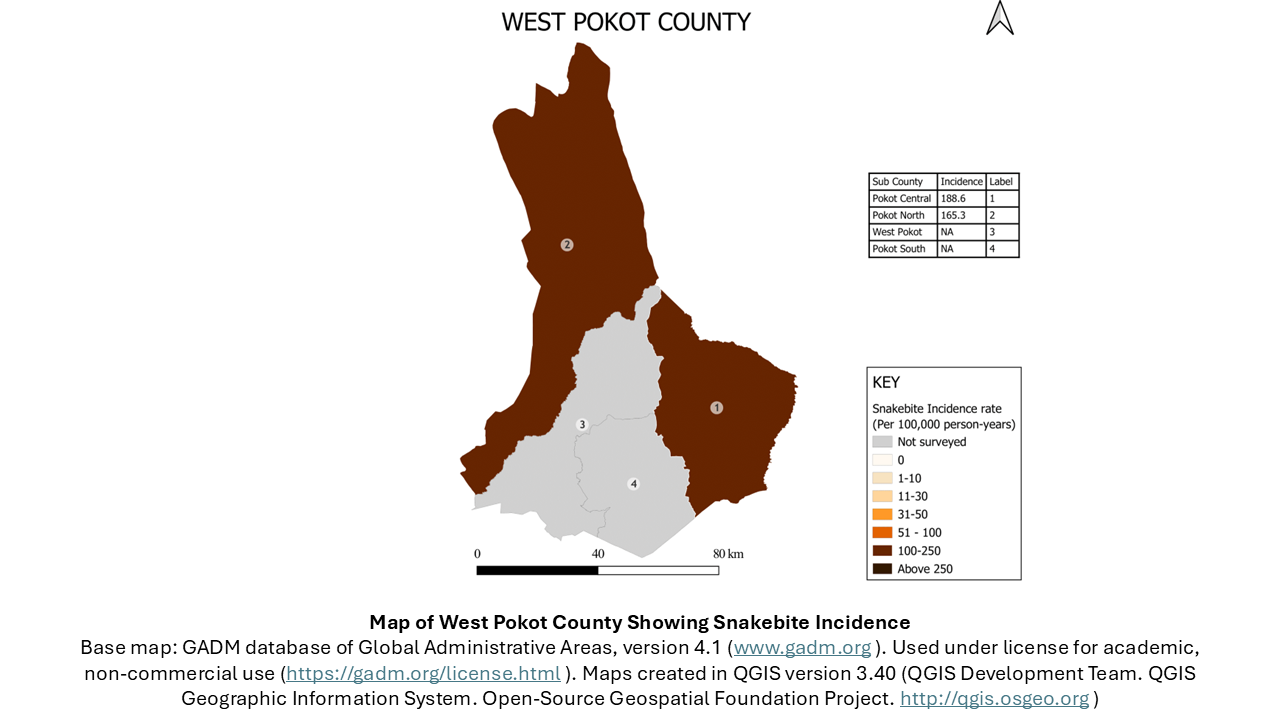

Supplement: S15 File — (TIF) [file pntd.0013732.s015.tif]
